# A polymorphism in ABCC4 is related to efficacy of 5-FU/capecitabine-based chemotherapy in colorectal cancer patients

**DOI:** 10.1038/s41598-017-07491-3

**Published:** 2017-08-01

**Authors:** Qi Chen, Fanyi Meng, Lei Wang, Yong Mao, Huan Zhou, Dong Hua, Hongjian Zhang, Weipeng Wang

**Affiliations:** 1 Center for Drug Metabolism and Pharmacokinetics, College of Pharmaceutical Sciences, Soochow University, Suzhou, China; 2 Department of Pharmacy, Jiangsu Cancer Hospital, Nanjing, China; 3 Department of Oncology, The Affiliated Hospital of Jiangnan University, Wuxi, China; 4 Department of Clinical Laboratory, The Affiliated Hospital of Jiangnan University, Wuxi, China

## Abstract

To investigate the association of microRNA (miRNA) binding-site polymorphisms in the drug transporter genes with the efficacy of 5-Fluorouracil (5-FU)/capecitabine-based chemotherapy in colorectal cancer (CRC), 6 polymorphisms were determined in 432 CRC patients by using DNA sequencing method. The impacts of the polymorphisms on the miRNA-mediated regulation of gene expression were evaluated by using the methods including quantitative real-time PCR, western blotting, and luciferase reporter assays. The effects of miRNA on the intracellular concentration and cytotoxicity of 5-FU in CRC cells were measured by high performance liquid chromatography conjected tandem mass spectrometry (HPLC-MS/MS) and MTT methods, respectively. Statistical analysis showed that a polymorphism rs3742106 in the 3′-UTR of ATP-binding cassette subfamily C member 4 (ABCC4) gene was significantly associated with the efficacy of 5-FU/capecitabine-based chemotherapy in CRC. The patients with T/T genotype had significantly higher response rate than those with G/G and G/T genotypes. The expression of ABCC4 was inhibited by miR-3190-5p through binding to the 3′-UTR of the ABCC4 gene. This regulatory role of miR-3190-5p was disrupted by rs3742106. Furthermore, we found that the intracellular concentration of 5-FU was elevated by miR-3190-5p, and consequently the sensitivity of CRC cells to 5-FU was also enhanced. Rs3742106 might be regarded as a genetic biomarker for individualized use of 5-FU and capecitabine in CRC.

## Introduction

Colorectal cancer (CRC) is one of the common digestive tract malignancies, and is the third most commonly diagnosed cancer and the fourth cause of cancer death worldwide^1^. There are more than one million new CRC cases and nearly 700 000 deaths worldwide in 2012^2^. In the United States, CRC is the third most common cancer, with more than 143,000 new cases and more than 52,000 deaths each year^3^. In addition, the incidence and mortality rates of CRC in Asia have increased rapidly over the past few decades^4, 5^. At present, treatments of CRC mainly include surgical treatment, radiation therapy, and chemotherapy. For chemotherapy, 5-FU and its prodrug capecitabine, oxaliplatin, and irinotecan are the first-line medicines used in clinic^6^. However, the response rates of 5-FU/capecitabine-based therapy are still very limited.

The biochemical pathway of 5-FU/capecitabine *in vivo* is well established. Upon absorption in the gut, capecitabine is metabolized to 5-FU *in vivo*, which then causes cytotoxicity by inhibiting synthesis of thymidine. In addition, 5-FU inhibits cell growth by converting to metabolites that are incorporated into DNA and RNA^7^. In the tumor cells, 5-FU is further metabolized to cytotoxic compounds to inhibit DNA synthesis by binding to thymidylate synthase. This pathway provides 25 candidate genes in which genetic variations might affect the efficacy of 5-FU/capecitabine^8^. Individual efficacy of 5-FU/capecitabine-based therapy may result from variable metabolism and transport of drugs and/or activated compounds caused by genetic variations. Dozens of publications exists regarding genetic biomarkers of individual efficacy of 5-FU/capecitabine-based therapy, but only a few have been identified with clinical significance^9^.

MicroRNA (miRNA) is a class of ~22 nts small endogenous noncoding RNA. MiRNA commonly inhibits gene expression by partially binding to the 3′-UTR of target genes. More than 1/3 gene expression are regulated by miRNAs, which are involved in cellular differentiation, proliferation, apoptosis and metabolism, and etc.^10^. Over the years, miRNAs have been discovered to play an important role in the expression of drug metabolism enzymes and transporters^11^. Numerous studies have shown that polymorphisms in the 3′-UTR of target genes lead to changes in miRNA regulatory roles and consequent gene expression levels^12^. However, the effects of the polymorphisms in the 3′-UTR of transporter genes on the efficacy of 5-FU/capecitabine-based therapy are still largely unknown.

In this study, we have investigated the relationship between the efficacy of 5-FU/capecitabine-based chemotherapy and the polymorphisms in the 3′-UTR of transporter genes. Our results demonstrate that a polymorphism rs3742106 in the 3′-UTR of ABCC4 gene is significantly related to the efficacy of 5-FU/capecitabine-based chemotherapy in CRC. The rs3742106 T allele offers a binding-site for miR-3190-5p, which results in low-expression of ABCC4, increased intracellular concentration of 5-FU, and enhanced sensitivity to 5-FU treatment.

## Results

### rs3742106 is related to therapy response in colorectal cancer

To investigate the relationship between the efficacy of 5-FU/capecitabine-based chemotherapy and the polymorphisms in the 3′-UTR of transporter genes, we determined the genotype distribution of six polymorphisms in 432 CRC patients by using DNA sequencing method. The typical genotyping results of the 6 polymorphisms are shown in Supplementary Fig. 1. As shown in Table 1, a polymorphism rs3742106 in the 3′-UTR of ABCC4 gene was in Hardy-Weinberg equilibrium and was significantly correlated to the efficacy of 5-FU/leucovorin/oxaliplatin (FOLFOX4) and capecitabine/oxaliplatin (XELOX) regimens. The response rates of both 5-FU- and capecitabine-based therapy in the T/T homozygous patients were apparently higher than those in the G/G homozygous patients (72.73% *vs* 49.18% for 5-FU; 67.27% *vs* 47.83% for capecitabine). The efficacy of 5-FU/capecitabine-based chemotherapy was not related to the polymorphisms rs562, rs1059751, rs2458225, rs3177514, and rs63282661 (data not shown). No correlation between the genotypes of the six polymorphisms and the occurrence of chemotherapy toxicity was observed (data not shown).

**Table 1:** The association of rs3742106 with the response of chemotherapy.

Chemotherapy	Genotype	R/D^a^	Efficacy (%)	OR (95% CI)	*P*-value for efficacy	*P*-value for HWE
FOLFOX4	G/G	30/31	49.18	1		0.43
	G/T	43/30	58.90	1.48 (0.75–2.94	0.298	
	T/T	24/9	72.73	2.76 (1.10–6.89)	0.031	
XELOX	G/G	44/48	47.83	1		0.33
	G/T	61/57	51.69	1.17 (0.68–2.02)	0.677	
	T/T	37/18	67.27	2.24 (1.12–4.50)	0.026	

^a^R, the number of patients achieving partial response and complete response; D, the number of patients with stable disease and progressive disease.

### rs3742106 influences miR-3190-5p-mediated inhibition of ABCC4 expression

**Figure 1:**
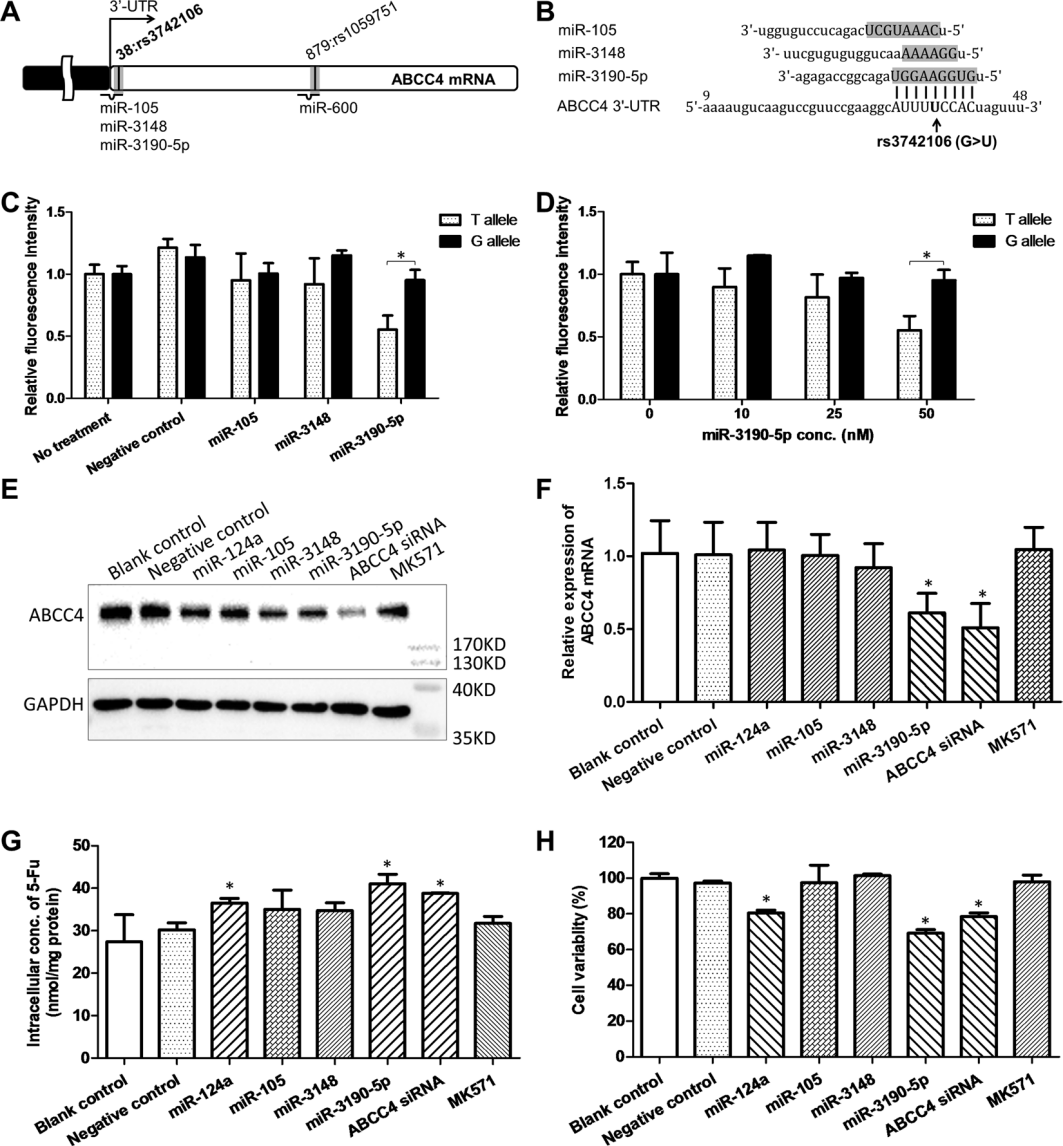
The effect of miR-3190-5p on the expression of ABCC4, transmembrane transportation of 5-FU, and proliferation of HCT-116 cells. (**A**) The polymorphisms in the predicted binding-sites of miRNAs in the 3′-UTR of ABCC4 gene. (**B**) The rs3742106 locates in the ‘seed-region’ of miR-105, miR-3148, and miR-3190-5p. The uppercase letters indicated the binding-sites for miRNAs. (**C**) Luciferase reporter analysis of the impact of miRNAs on the expression of the T- and G-allelic ABCC4/3′-UTR/pGL3 constructs. (**D**) Luciferase reporter analysis of the impact of miR-3190-5p on the expression of the T- and G-allelic ABCC4/3′-UTR/pGL3 constructs. (**E**) The effect of miR-124a, miR-105, miR-3148, miR-3190-5p, ABCC4 siRNA, and MK571 on the expression of ABCC4 protein in HCT-116 cells. The full-length blots are presented in Supplementary Fig. 4. (**F**) The effect of miR-124a, miR-105, miR-3148, miR-3190-5p, ABCC4 siRNA, and MK571 on the expression of ABCC4 mRNA in HCT-116 cells. (**G**) The effect of miR-124a, miR-105, miR-3148, miR-3190-5p, ABCC4 siRNA, and MK571 on the intracellular concentration of 5-FU in HCT-116 cells. In Figures E–G, the cells were treated with the regulators for 72 h before the addition of 5-FU. (**H**) The effect of miR-124a, miR-105, miR-3148, miR-3190-5p, ABCC4 siRNA, and MK571 on the growth of HCT-116 cells co-treated with 5-FU. Each experiment was repeated for three times; the data were presented in the histograms as mean with SD, and were statistically analyzed by using *t*-test as compared with the negative control; *P < 0.05.

To explore the potential function of rs3742106, we used the bioinformatics software miRanda to predict miRNAs that might bind to the 3′-UTR of the ABCC4 gene. We found that rs3742106 located in the binding-sites of miR-105, miR-3148, and miR-3190-5p (Fig. 1A and B). Therefore, we constructed rs3742106 T- and G-allelic ABCC4/3′-UTR/pGL-3 constructs, and then transfected them into CHO cells with miR-105, miR-3148, or miR-3190-5p mimics. We found that the expression of T-allelic recombinant plasmids was significantly suppressed by miR-3190-5p mimics (*P* < 0.05) but not by miR-105 and miR-3148 mimics (Fig. 1C). However, the expression of the G-allelic recombinant plasmids was not impacted by miR-105, miR-3148, and miR-3190-5p mimics (Fig. 1C). The miR-3190-5p-mediated inhibition of the expression of T-allelic recombinant plasmids was in a dose-dependent manner (Fig. 1D). Then we evaluated the regulatory role of miR-105, miR-3148, and miR-3190-5p mimics on the expression of ABCC4 protein and mRNA in the TT homozygous HCT-116 cells. MiR-124a, a miRNA can inhibit the expression of ABCC4^13^, and MK571, a specific inhibitor of ABCC4, as well as ABCC4 siRNA were used as positive controls. We found that the expression of both ABCC4 protein and mRNA was apparently inhibited by miR-3190-5p mimics and ABCC4 siRNA, but not by miR-105 and miR-3148 mimics (Fig. 1E and F). The expression of ABCC4 protein instead of mRNA was suppressed by miR-124a mimics (Fig. 1E and F).

### miR-3190-5p augments intracellular concentration of 5-FU

ABCC4 is an efflux transporter that is capable of transporting a range of endogenous substances, metabolites and drugs out of cells. Since miR-3190-5p can inhibit the expression of ABCC4, we investigated the effect of miR-3190-5p on the ABCC4-mediated transportation of 5-FU. We treated HCT-116 cells with miR-124a mimics, miR-3148 mimics, miR-3190-5p mimics, ABCC4 siRNA, or MK571 for 72 h, and then added 10 mM of 5-FU to the medium. Two hours later, we determined the intracellular concentration of 5-FU by using HPLC-MS/MS method (Supplementary Fig. 2). We found that the intracellular concentration of 5-FU was markedly augmented upon the treatment of miR-124a mimics, miR-3190-5p mimics, and ABCC4 siRNA (Fig. 1G).

### miR-3190-5p enhances cell sensitivity to 5-FU

To explore the effect of miR-3190-5p on cell sensitivity to 5-FU, we treated HCT-116 cells with 50 nM of miR-3190-5p mimics and 5 μM of 5-FU. MiR-124a mimics, ABCC4 siRNA, and MK571 were used as positive controls. We found that the 5-FU-mediated inhibition of the cell growth was further enhanced by miR-124a mimics, miR-3190-5p mimics, and ABCC4 siRNA (Fig. 1H). These findings suggest that the sensitivity of HCT-116 cells to 5-FU can be improved by miR-124a mimics, miR-3190-5p mimics, and ABCC4 siRNA, which can inhibit the expression of ABCC4 protein (Fig. 1E) and elevate the intracellular concentration of 5-FU (Fig. 1G).

## Discussion

**Figure 2:**
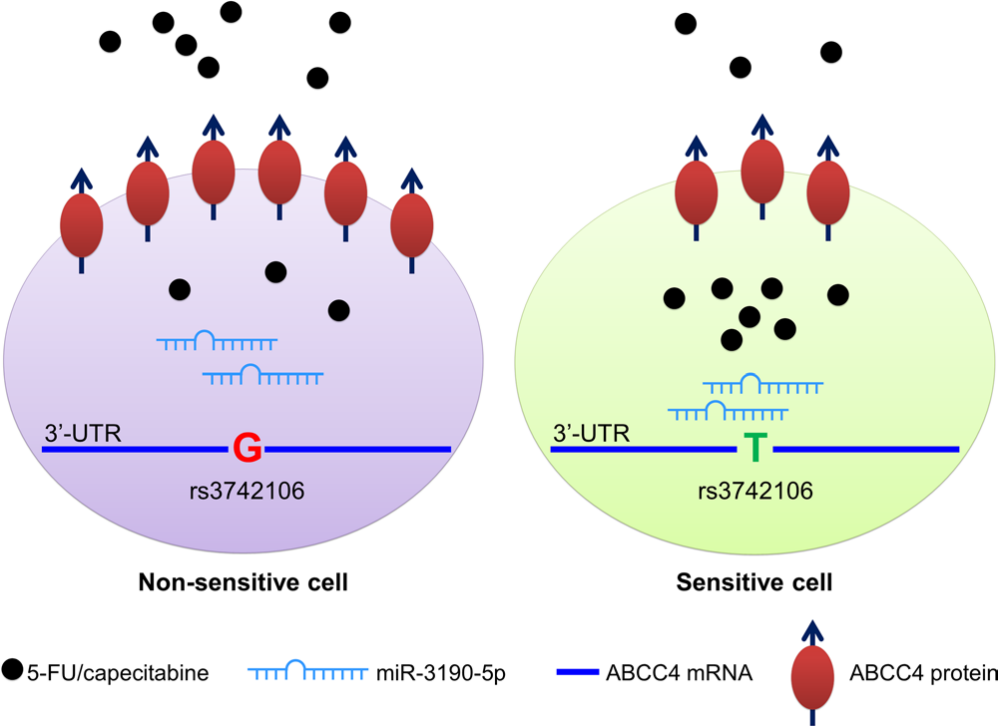
The molecular mechanism of individual response to chemotherapy. Rs3742106 G-allelic ABCC4 disrupts the binding of miR-3190-5p and results in over-expression of ABCC4 and consequent low intracellular concentration of drug, as well as resistance to chemotherapy. On the contrary, the T-allelic ABCC4 generates a binding-site for miR-3190-5p and leads to attenuated expression of ABCC4 protein, elevated intracellular concentration of 5-FU, and increased sensitivity of cells to the chemotherapy.

In this study, we find that a polymorphism rs3742106 in the 3′-UTR of ABCC4 gene is significantly related to the efficacy of 5-FU and capecitabine-based chemotherapy in CRC. The patients with rs3742106 T/T genotype are much more sensitive to the therapy of 5-FU and capecitabine than those with G/G genotype. Rs3742106 T-allelic 3′-UTR generates a binding-site for miR-3190-5p and leads to attenuated expression of ABCC4 protein and elevated intracellular concentration of 5-FU, as well as improved sensitivity of CRC cells to the chemotherapy of 5-FU (Fig. 2).

Increasing evidence shows the importance of miRNA-related polymorphisms in the chemotherapy of cancer. These polymorphisms impact the regulatory role of miRNAs and consequently affect the expression of target genes, leading to individual response to chemotherapy in various cancers including CRC. For instance, a polymorphism 829 C > T in the 3′-UTR of dihydrofolate reductase (DHFR) gene disrupts the binding of miR-24, resulting in increased expression of DHFR and consequent methotrexate resistance^14^. It is also reported that *let*-7 inhibits the expression of KRAS (KRAS proto-oncogene, GTPase) by binding to its 3′-UTR. In the patients with metastatic CRC, a polymorphism rs1764370 in KRAS leads to loss function of *let*-7, increased expression of KRAS, and drug resistance of cetuximab-irinotecan chemotherapy, as well as reduced overall survival and progression-free survival^15^. A polymorphism C421A in ATP binding cassette subfamily G member 2 (ABCG2) gene affects the expression level of ABCG2 protein and individual efficacy of antineoplastic drugs through influencing the regulatory role of hsa-miR-519c and hsa-miR-328^16^. In this study, we discover a novel polymorphism rs3742106 in the 3′-UTR of ABCC4 gene leads to individual efficacy of 5-FU- and capecitabine-based chemotherapy in CRC. This polymorphism affects the expression of ABCC4 protein on cancer cells through altering the inhibitory role of miR-3190-5p.

ABCC4, also known as the multi-drug resistance-associated protein 4, is an important member of the ATP-binding cassette transporter family. ABCC4 is capable of transporting a variety of endogenous and exogenous organic anions with different constructions out to cell. ABCC4 is also a versatile efflux transporter for many drugs, especially antiviral drugs, antitumor drugs, and diuretics^17^. ABCC4 confers cells resistance to cytotoxic complexes, protects important tissues from aberrant biological damage and at the same time affects drug metabolism in cells, resulting in drug resistance. Significant association between downregulation of ABCC4 and sensitivity to neoadjuvant chemo-radiotherapy, as well as between low ABCC4 expression and longer disease-free survival in rectal cancer patients has been demonstrated^18, 19^, indicating that ABCC4 might be a possible predictive biomarker for the efficacy of chemotherapy in CRC. Recent reports demonstrate that the expression of ABCC4 can be attenuated by miR-124a, miR-125a, miR-125b, miR-143, and miR-506^13, 20^. In the current study, miR-105, miR-3148, and miR-3190-5p are also predicted *in silico* to bind to the 3′-UTR of ABCC4 but only miR-3190-5p is observed to repress endogenous expression of ABCC4 protein and mRNA in CRC cells. This might because that there are nine complementary bases in the binding-site of miR-3190-5p, but only eight and six bases for miR-105 and miR-3148, respectively. Furthermore, luciferase reporter assays confirm that miR-3190-5p directly recognizes the rs3742106 T-allelic 3′-UTR of ABCC4 and inhibits its expression in a dose-dependent manner. Taken together, these findings provide evidences to support the conclusion that miR-3190-5p plays a significant role in inhibiting the translation of ABCC4.

Recent studies demonstrate that ABCC4 expression is elevated in 5-FU-resistant cells^21^, and the sensitivity of cells can be enhanced through inhibiting the expression of ABCC4 by siRNA^22^. In addition, miR-320a and miR-4496 have been revealed to decrease CagA induced chemo-resistance by targeting efflux transporter ABCG2 at the transcriptional and post-transcriptional level, respectively. Combination therapy of 5-FU with miR-320a/-4496 inhibited gastric tumorigenesis and metastatic potential in mice^23^. Furthermore, miR‑302a enhances 5‑FU‑induced cell death in human colon cancer cells through inhibiting the expression of insulin like growth factor 1 receptor (IGF1R)^24^. Therefore it’s possible that the sensitivity of cancer cells to 5-FU might also be enhanced by miR-3190-5p, which is able to suppress the expression of ABCC4. Indeed, we found that the inhibitory rate of 5-FU in HCT-116 cells was significantly elevated by miR-3190-5p mimics (Fig. 1H), indicating a synergistic action of miR-3190-5p and 5-FU in anti-tumor. These findings suggest a potential combination therapy of 5-FU with miR-3190-5p in rs3742106 TT homozygous CRC patients.

Except for miR-3190-5p, miR-124a and ABCC4 siRNA are also found to inhibit the expression of ABCC4 protein, but only miR-3190-5p and ABCC4 siRNA are able to repress the expression of ABCC4 mRNA. These results indicate that miR-3190-5p and ABCC4 siRNA can inhibit the expression of ABCC4 at both post-transcriptional and translational level, but miR-124a suppresses ABCC4 expression only at translational level. Since miR-3190-5p, miR-124a and ABCC4 siRNA can inhibit the expression of ABCC4 protein, these small RNAs are observed to increase the intracellular concentration of 5-FU and improve the sensitivity of cancer cells to 5-FU treatment. Since the intracellular concentration of 5-FU is elevated by MK571 in 12 h and then reduced to baseline level in 24 h (Supplementary Fig. 3), the sensitivity of cancer cells to 5-FU treatment is not enhanced by MK571 after incubation for 72 h. This might because of a reversible binding of MK571 to ABCC4 and/or degradation of MK571 in cell culture.

In summary, we provide evidences to support the conclusion that a polymorphism rs3742106 in the 3′-UTR of ABCC4 through altering the binding of miR-3190-5p to ABCC4 mRNA leads to disrupted expression of ABCC4, intracellular concentration of 5-FU, and individual response to 5-FU and capecitabine chemotherapy in CRC. In the patients with rs3742106 T/T genotype, the expression of ABCC4 on cancer cells is suppressed by miR-3190-5p, thus the efflux of 5-FU is reduced and the sensitivity to 5-FU is enhanced. While in the patients with G/G genotype, ABCC4 is overexpressed on cancer cells and intracellular concentration of 5-FU is decreased, so that the drug resistance is elevated. These findings provide a novel molecular marker for individual chemotherapy of 5-FU- and capecitabine-based in CRC, as well as the new regulatory mechanism of polymorphism and miRNA in the expression of ABCC4.

## Materials and Methods

### Patients

The research protocol was approved by the institutional review board of Soochow University. All experiments were performed in accordance with the relevant guidelines and regulations of Soochow University. A total of 432 CRC patients participated in this study after acquisition of informed consent. They were recruited from the Affiliated Hospital of Jiangnan University (Wuxi, Jiangsu province) and Jiangsu Cancer Hospital (Nanjing, Jiangsu province) in recent 3 years. The characteristics of the patients are listed in Supplementary Table 1. 167 CRC patients received FOLFOX4 regimen (5-FU + oxaliplatin + calcium folinate) chemotherapy, and 265 patients received the chemotherapy of XELOX regimen (capecitabine + oxaliplatin). The efficacy of chemotherapy including complete response, partial response, stable disease, and progressive disease was evaluated according to post-treatment CT results. The toxicity symptoms of chemotherapy were recorded, including nausea and vomiting, diarrhea, mucositis, stomatitis, neutropaenia, and thrombocytopaenia.

### Genotyping

The whole bloods from the patients were collected in EDTA anticoagulant tubes (VacutainerR, BD), from which the genomic DNAs were extracted by using phenol/chloroform method. The purity and concentration of the DNAs were determined by using UV-Vis spectrophotometer. The polymorphisms in the 3′-UTR of the transporter genes were obtained from the online database miRNASNP 2.0 (www.bioguo.org/miRNASNP2). Six polymorphisms with minor allele frequencies (MAF) of not less than 10% were investigated in this study (Supplementary Table 2). The genotypes of the candidate polymorphisms were determined by using DNA sequencing method with the primers in (Supplementary Table 3).

### Cells

The CHO and HCT-116 cells were purchased from AmericanType Culture Collection (Manassas, VA). They were cultured in DMEM or RPMI 1640 medium (Hyclone) containing 10% fetal bovine serum (FBS; GIBCO) at 37 °C in 5% CO_2_. Cells in the logarithmic growth phase were used for experiments.

### Luciferase reporter assay

The rs3742106 G-allelic ABCC4/3′-UTR/pGL-3 constructs were generated as before^25^. The 3′-UTR of ABCC4 gene was amplified with the primers 5′-pCTA GAT CAA GTC CGT TCC GAA GGC ATT TGC CAC TT-3′ (forward) and 5′-pCTA GAA GTG GCA AAT GCC TTC GGA ACG GAC TTG AT-3′ (reverse). The products were digested by restriction endonucleases *Xba*I and *Hpa*I, and then were cloned into pGL3-control vector (Promega). The T-allelic ABCC4/3′-UTR/pGL-3 constructs were generated by using site-directed mutagenesis PCR method with the primers 5′-pCTA GAT CAA GTC CGT TCC GAA GGC ATT TTC CAC TT-3′ (forward) and 5′-pCTA GAA GTG GAA AAT GCC TTC GGA ACG GAC TTG AT-3′ (reverse) as described before^25^. The recombinant plasmids were validated by PCR, endonucleases digestion, and DNA sequencing methods. The ABCC4/3′-UTR/pGL-3 constructs were co-transfected into the CHO cells with pRL-TK plasmid (Promega), miR-3148, miR-3190-5p and miR-105 mimics (GenePharma Inc. Shanghai, China), or an unrelated miRNA sequence as negative control. After 24 h of incubation, the luciferase activities were analyzed by using the dual-luciferase reporter assay system (Promega).

### Western blot

Protein concentrations were measured using the Pierce BCA Protein Assay Kit (Thermo) according to the manufacturer’s instructions. Twenty μg of protein were separated on 10% SDS-PAGE gel and electro-transferred onto NC membranes. The membranes were incubated with ABCC4 or GAPDH mouse monoclonal antibodies (Santa Cruz Biotech, USA) and subsequently with a peroxidase goat anti-rabbit IgG (Santa Cruz Biotech). The membranes were then developed with Clarity Western ECL substrates (Merck Millipore) and visualized with the ChemiDocTM MP Imaging System (Bio-Rad).

### Quantitative real-time PCR

Total RNAs from the HCT-116 cells transfected with miR-124a, miR-3148, miR-3190-5p, ABCC4 siRNA, and MK571 were isolated using TRIzol (Invitrogen). The RNAs were then reversely transcripted using M-MuLV reverse transcriptase (MBI) and random primer (Sangon Biotech., Shanghai, China). Quantitative real-time PCRs (qPCRs) were conducted in 20-μL reaction containing quantitative RT-PCR master mix (Takara), primers 5′-GGA TCC AAG AAC TGA TGA GTT AAT-3′ (forward) and 5′-TCA CAG TGC TGT CTC GAA AAT AG-3′ (reverse) for ABCC4, or 5′-TGC ACC ACC AAC TGC TTA GC-3′ (forward) and 5′-GGC ATG GAC TGT GGT CAT GAG-3′ (reverse) for GAPDH. GAPDH was used as internal standard. The qPCRs were carried out on the CFX96 Touch^TM^ real-time PCR system (Bio-Rad). The expression levels were calculated by using the comparative threshold cycle (Ct) method with the formula 2^−∆∆Ct^.

### Quantitation of intracellular drug by LC-MS/MS

To investigate the effect of miR-3190-5p on the transport of 5-FU, we first transfected HCT-116 cells with miR-3190-5p mimics for 72 h, and then added 5-FU to the medium. Two hours later, intracellular concentration of 5-FU was measured by a LC–MS/MS system consisting of an API4000 Qtrap mass spectrometer equipped with a turbo-V ionization source (Applied Biosystems, Foster City, CA, USA), two LC-20AD pumps with a CBM-20A controller, DGU-20A solvent degasser, and a SIL-20A autosampler (Shimadzu, Columbia, MD, USA). An AgelaVenusil XBP C_18_ column (50 × 2.1 mm, 5 μm) was used. Column temperature was held at 40 °C. The mobile phase consisted of water (A) and methanol (B). Gradient elution was performed as follows: 0–0.5 min, 5% B; 0.5–1 min, 5–95% B; 1–4.5 min, 95% B; 4.5–4.6 min 95–5% B; 4.6–6 min 5% B. The mass spectrometer was operated in the electron spray ionization negative mode with multiple reaction-monitoring. Both 5-FU and the internal standard (betamethasone) were monitored with a dwell time set at 100 ms. The MS/MS parameters were set as follows: curtain gas, 30 psi; nebulizer gas, 55 psi; turbo gas, 55 psi; ion spray voltage, 4500 V; and ion source temperature, 450 °C. In the selected ion transitions, collision energy and declustering potential values were optimized as −46 and −30 V for 5-FU, −110 and −10 V for betamethasone, respectively. Ion transitions were monitored at 129.9 → 42.1 m/z for 5-FU and 391.1 → 361.4 m/z for betamethasone.

### MTT assay

The effect of miRNAs on cell sensitivity to 5-FU was determined by MTT assay. The HCT-116 cells were seeded in 96-well culture plates at 5000 cells/well. Twenty-four hours later, 50 nM of miRNA mimics were transfected into the cancer cells using lipofectamine 2000. Lipofectamine 2000 only or with a scramble miRNA was used as blank control and negative control, respectively. miR-124a, a regulatory miRNA for ABCC4^13^, ABCC4 siRNA (GenePharma Inc. Shanghai, China), and a specific ABCC4 inhibitor MK571 were used as positive controls. Twenty-four hours later, 5 μM of 5-FU was directly added into culture medium. Fourty-eight hours later, the cells are incubated with MTT (Sigma) for 2 h. Then the medium was replaced with 150 μl of dimethyl sulphoxide (Merck) and oscillated at 37 °C for 10 min. The absorbance at 490 nm was measured using the M3 SpectraMax microplate reader. The effect of miRNAs on cell growth was estimated according to the absorbance.

### Statistical analysis

The association between genotypes and the efficacy of chemotherapy were assessed by using Chi-square test. The odds ratios (ORs) and 95% confidence intervals (CIs) were obtained using Unconditional Univariate Logistic Regression model. Hardy–Weinberg equilibrium (HWE) of the genotype distribution among patients was analyzed using a goodness-of-fit chi-square test. All of the statistical analyses were carried out by two analysts independently in a blind fashion using SPSS11.5 software. *P* < 0.05 was considered statistically significant.

## Electronic supplementary material

Supplementary Information
