# Supplementary material for: A polymorphism in ABCC4 is related to efficacy of 5-FU/capecitabine-based chemotherapy in colorectal cancer patients
Source: Sci Rep. 2017 Aug 1;7:7059. doi: 10.1038/s41598-017-07491-3 (PMC5539293; doi:10.1038/s41598-017-07491-3)
Supplement: Supplementary file 1 — Supplementary Information [file 41598_2017_7491_MOESM1_ESM.doc]

**A polymorphism in ABCC4 is related to efficacy of 5-FU/capecitabine-based chemotherapy in colorectal cancer patients**

Qi Chen^a,#^, Fanyi Meng^a,#^, Lei Wang^b^,*, Yong Mao^c^, Huan zhou^d^, Dong Hua^c^, Hongjian Zhang^a^, Weipeng Wang^a^,*

**Supplementary materials**

**Supplementary Table 1.** The characteristics of the patients.

| Characteristics | Variable | XELOX group | FOLFOX4 group |
| --- | --- | --- | --- |
| Gender | female | 100 (37.74%) | 68 (40.72%) |
|  | male | 165 (62.26%) | 99 (59.28%) |
| Age (year) | ≤60 | 158 (59.62%) | 99 (59.28%) |
|  | >60 | 107 (40.38%) | 68 (40.72%) |
| Tumor size>5cm | no | 172 (64.91%) | 132 (79.04%) |
|  | yes | 93 (35.09%) | 35 (20.96%) |
| Depth of tumor infiltration | T1 | 2 (0.75%) | 5 (2.99%) |
|  | T2 | 27 (10.19%) | 17 (10.18%) |
|  | T3 | 151 (56.98%) | 95 (56.89%) |
|  | T4 | 85 (32.08%) | 50 (29.94%) |
| Lymph node metastasis | no | 98 (36.98%) | 67 (40.12%) |
|  | yes | 167 (63.02%) | 100 (59.88%) |
| Distant metastasis | no | 196 (73.96%) | 128 (76.65%) |
|  | yes | 69 (26.04%) | 39 (23.35%) |
| Pathological type | infiltrating type | 25 (9.43%) | 17 (10.18%) |
|  | protruded type | 89 (33.58%) | 32 (19.16%) |
|  | ulcerative type | 128 (48.30%) | 104 (62.28%) |
|  | other | 23 (8.68%) | 14 (8.38%) |
| Differentiation grade | well | 12 (4.53%) | 2 (1.20%) |
|  | moderate | 216 (81.51%) | 138 (82.63%) |
|  | poor | 37 (13.96%) | 27 (16.17%) |
| TNM stage | I | 11 (4.15%) | 10 (5.99%) |
|  | II | 72 (27.17%) | 45 (26.95%) |
|  | III | 117 (44.15%) | 70 (41.92%) |
|  | IV | 65 (24.53%) | 42 (25.15%) |
| Tumor type | colon | 146 (55.09%) | 82 (49.10%) |
|  | rectal | 119 (44.91%) | 85 (50.90%) |
| Efficacy | complete response | 11 (4.15%) | 5 (2.99%) |
|  | partial response | 94 (35.47%) | 92 (55.09%) |
|  | stable disease | 51 (19.25%) | 35 (20.96%) |
|  | progressive disease | 109 (41.13%) | 35 (20.96%) |
| Adverse events | no | 93 (35.09%) | 72 (43.11%) |
|  | yes | 172 (64.91%) | 95 (56.89%) |

**Supplementary Table 2.** Polymorphisms in the 3’-UTR of the transporter genes.

| SNP | Variation | MAF^a^ | Gene | miRNA | Effect on binding |
| --- | --- | --- | --- | --- | --- |
| rs3742106 | T>G | G=0.3976 | ABCC4 | miR-105 | gain |
|  |  |  |  | miR-3148 | loss |
|  |  |  |  | miR-3190-5p | loss |
| rs1059751 | T>C | C=0.4273 | ABCC4 | miR-600 | loss |
| rs562 | C>T | T=0.4813 | ABCC5 | miR-4272 | gain |
| rs2458225 | T>C | C=0.4771 | SLC28A2 | miR-3622b-5p | gain |
| rs63282661 | A>G | G=0.4374 | SLC29A2 | miR-1281 | loss |
| rs3177514 | C>A | A=0.4365 | SLC29A2 | miR-1207-3p | loss |
|  |  |  |  | miR-744 | gain |
|  |  |  |  | miR-4283 | gain |

^a^ MAF, minor allelic frequency in 1000 Genomes Project (<http://www.internationalgenome.org/>).

**Supplementary Table 3.** The primers used for genotyping.

| SNP | Primer sequence (5’→3’) ^a^ | Product size (bp) |
| --- | --- | --- |
| rs562 | F: AAGGCCCACAGAGATGATTC  R: TTCCCACACCTCCACAGTTC | 572 |
| rs1059751 | F: TGTAGTAGGGAGTGTGTACC  R: AGGTCATGAGACTCCACTTC | 711 |
| rs2458225 | F: CAATACCGTCTGTGCCTAAG  R: GAGTAGCTGGGACTACAGTC | 335 |
| rs3177514 | F: GAGGAACAGATGTGGAGGAC  R: TCTACCACGGACCAGTCACT | 299 |
| rs3742106 | F: GCAACTTCACAGTCTTCAAG  R: CAGGTTGTTTGGCATTCAGC | 730 |
| rs63282661 | F: AGCTGCTCTTCATCCACTTG  R: AGTGCAGTACACACATGCAG | 458 |

^a^ F, forward; R, reverse.


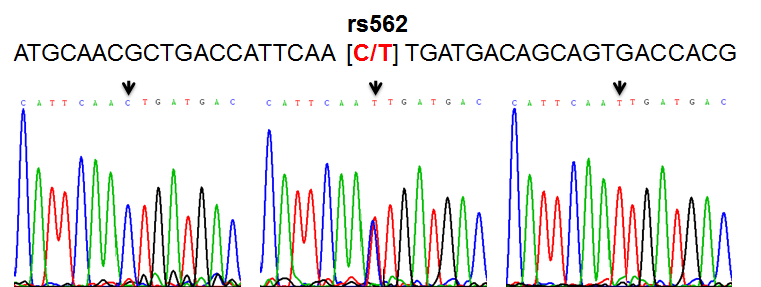


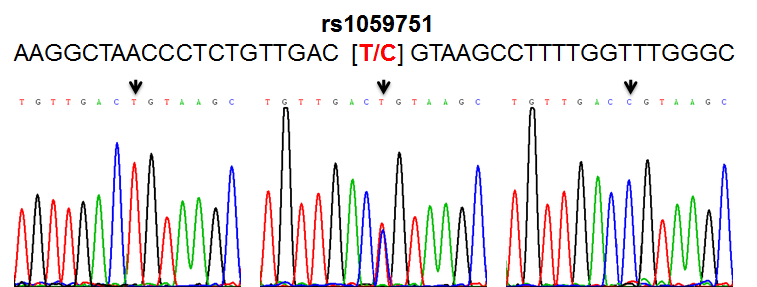


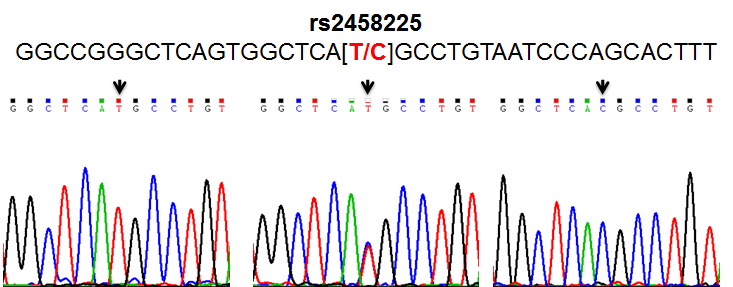


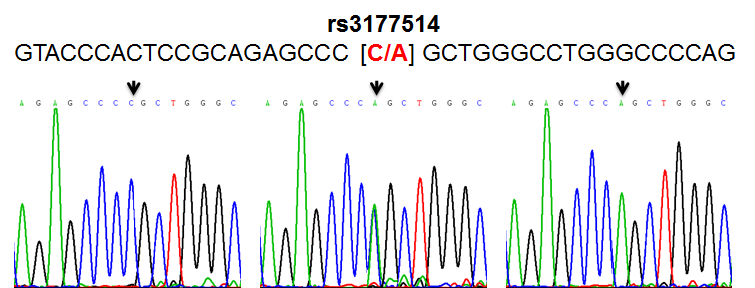


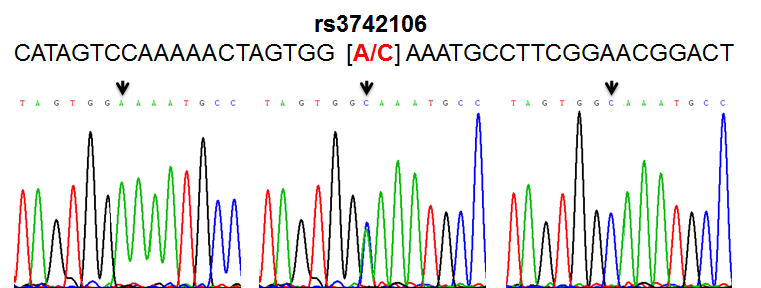


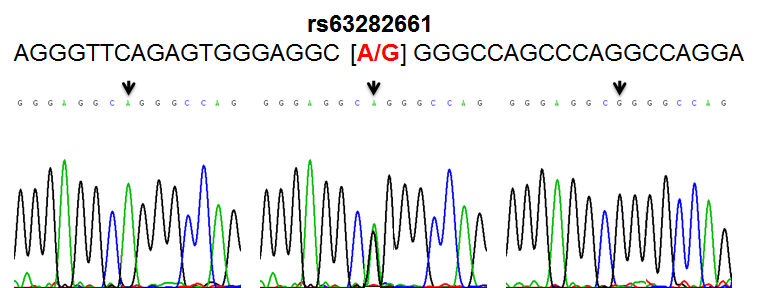


**Supplementary Figure 1.** The typical genotyping results.

**Supplementary Figure 2.** The chromatogram and mass spectrum of 5-FU determined by LC-MS/MS method. (A) The representative total ion chromatogram; (B) The molecular ion mass spectrum of 5-FU; (C) The secondary ion mass spectrum of 5-FU.

**Supplementary Figure 3**. The intracellular concentration of 5-FU in HCT-116 cells treated with 10 μM MK571 for 0 h, 2 h, 12 h, 24 h, 48 h, and 72 h, respectively.

**
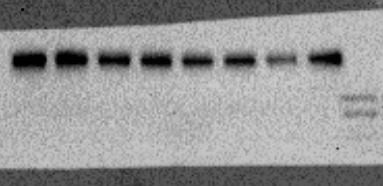
**

**
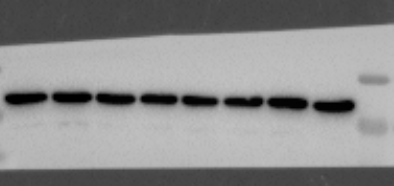
**

**Supplementary Figure 4.** The full-length blots used in Fig. 1E.
